# Evaluating the Effect of Cognitive Dysfunction on Mental Imagery in Patients with Stroke Using Temporal Congruence and the Imagined ‘Timed Up and Go’ Test (iTUG)

**DOI:** 10.1371/journal.pone.0170400

**Published:** 2017-01-26

**Authors:** Maxime Geiger, Céline Bonnyaud, Yves-André Fery, Bernard Bussel, Nicolas Roche

**Affiliations:** 1 Inserm Unit 1179, Team 3: Technologies and Innovative Therapies Applied to Neuromuscular diseases, UVSQ, CIC 805, Physiology–Functional Testing Ward, AP-HP, Raymond Poincaré Teaching Hospital, Garches, France; 2 STAPS Department of Versailles, Versailles University of Saint-Quentin- en-Yvelines, Saint-Quentin- en-Yvelines, France; "INSERM", FRANCE

## Abstract

**Background:**

Motor imagery (MI) capacity may be altered following stroke. MI is evaluated by measuring temporal congruence between the timed performance of an imagined and an executed task. Temporal congruence between imagined and physical gait-related activities has not been evaluated following stroke. Moreover, the effect of cognitive dysfunction on temporal congruence is not known.

**Objective:**

To assess temporal congruence between the Timed Up and Go test (TUG) and the imagined TUG (iTUG) tests in patients with stroke and to investigate the role played by cognitive dysfunctions in changes in temporal congruence.

**Methods:**

TUG and iTUG performance were recorded and compared in twenty patients with chronic stroke and 20 controls. Cognitive function was measured using the Montreal Cognitive Assessment (MOCA), the Frontal Assessment Battery at Bedside (FAB) and the Bells Test.

**Results:**

The temporal congruence of the patients with stroke was significantly altered compared to the controls, indicating a loss of MI capacity (respectively 45.11 ±35.11 vs 24.36 ±17.91, p = 0.02). Furthermore, iTUG test results were positively correlated with pathological scores on the Bells Test (r = 0.085, p = 0.013), likely suggesting that impairment of attention was a contributing factor.

**Conclusion:**

These results highlight the importance of evaluating potential attention disorder in patients with stroke to optimise the use of MI for rehabilitation and recovery. However further study is needed to determine how MI should be used in the case of cognitive dysfunction.

## 2 Background

In clinical practice, functional motor activity is routinely assessed using the ‘Timed Up and Go’ (TUG) test. The TUG test involves the patient standing up from a chair, walking 3m, turning around, walking 3m back to the chair and sitting down [1]. It has been validated for use in patients with stroke and several studies have shown that TUG test performance is altered in these patients [2–4] [5,6]. Cognitive dysfunction, which is common after stroke, may have an impact on motor capacity and can interfere with gait-related activities. Correlations have been found between cognitive impairments and activity restrictions in stroke patients [5,7]. Several studies also showed that performance on the TUG test is altered in subjects with cognitive impairments compared with matched, cognitively intact subjects (patients with stroke and elderly patients) [5,6].

The relationship between cognitive function and motor activity can be investigated using Motor Imagery (MI). MI is a cognitive state that corresponds to imitating or anticipating the effects or memory of an action [8]. MI activates the same neurones that are involved in processes that precede actual movement and actions [8–10], and can be experienced in two ways: (1) Kinesthetic MI (or KI), which involves imagining, to “feel” the sensation of the movement (muscle contractions and muscle stretch) during the mental imagery or (2) visual MI (VI) during which patients imagine themselves performing the action either in the first-person (internal VI) or the third-person (external VI) [11–13].

The difference between the time taken to perform a real action (e.g. TUG) and the time to imagine the same action (e.g. **imagined** TUG) is termed temporal congruence. For the TUG test, it is calculated as follows: [(TUG-iTUG)/((TUG+iTUG)/2)*100] [14]. In healthy controls, the time taken to imagine an action is practically the same as the actual time taken to perform that action, i.e. TUG ≈ iTUG [15–17]. Research has revealed a weak temporal congruence between the time to carry out a task through MI and time to physically carry out the task in patients with stroke, although the direction of the difference is not clear. Some studies of upper limb tasks have reported that the time taken to imagine a task was longer than the time to carry out the real task, with large differences between the durations of executed and imagined movements) [8,18,19], while other studies found activities like eating with a spoon, hair combing or page turning were performed faster using MI than in reality [20].

To our knowledge, only a few studies have assessed the temporal congruence of lower limb tasks in patients with stroke[18,21,22], and only one has assessed gait related activities (walking forwards and backwards and side-stepping) [23]. Malouin et al (2004;2012) found a weaker temporal congruence for voluntary lower limb tasks in patients with right hemispheric lesions [18,21]. Similar to the results obtained with upper limb MI tasks, they found an alteration of temporal congruence between imagined and executed movement, demonstrating alterations in MI capacity in this population for voluntary lower limb movements, particularly in patients with right hemispheric lesions. Recently, Fusco et al., (2016) assessed MI in twelve patients with stroke. They asked the patients to perform mental representations of different walking tasks (forwards, backwards and side-stepping) accompanied or not by movements imitating walking. These authors found a more marked alteration of temporal congruence when patients performed the MI tasks without performing simultaneous imitating movements, this paradigm is slightly different from those used in the present study nevertheless it is interesting since it is the only one which assessed also gait related activities.

In addition, Beauchet et al (2010) used the TUG test to assess temporal congruence in elderly patients and found a greater error in temporal congruence (demonstrating a weaker imagery capacity) in older adults compared to younger adults [14]. A relationship was also found between increased temporal congruence and the Mini Mental State Evaluation (MMSE) score, suggesting cognitive impairments contributed to the differences in temporal congruence. Allali et al (2012) drew similar conclusions in patients with Multiple Sclerosis, reporting that temporal congruence between the TUG and iTUG was related to cognitive functions like attention, memory and executive functions [24].

To our knowledge, temporal congruence between the imagined and real Timed up and go (TUG), which is composed of several different types of gait related activities, has not yet been evaluated following stroke. The aim of this study was therefore to investigate temporal congruence between the TUG and iTUG in patients with stroke and in healthy controls. The second aim was to evaluate the effect of cognitive dysfunction on temporal congruence since the movements that compose the TUG are both semi-automatic and voluntary, and involve different central nervous processes.

We postulated that (i) temporal congruence between the TUG and iTUG would be weaker in patients with stroke than healthy controls (ii) MI capacity would be reduced by cognitive impairment in the domains of attention, memory and/or executive functions (as suggested by the results of Beauchet and Allali).

## 3 Methods

Twenty patients with stroke and twenty control subjects were included in this study (see Table 1). The control group were volunteers with no history of neurological or orthopaedic pathology that could interfere with the task, they were recruited from the staff of the University Hospital. Patients were recruited from the University Hospital during routine consultations and were eligible for inclusion if they: i) were over eighteen years old, ii) had hemiparesis due to a single stroke more than six months previously, iii) were able to perform the TUG test independently with or without walking aids. Exclusion criteria included: i) bilateral cortical lesions, ii) cerebellar syndrome, iii) severe comprehensive deficit or severe aphasia (score = 0 on the SOFMER’ scale of aphasia severity), iv) apraxia and v) musculoskeletal surgery less than six months previously. All subjects gave written consent before participation. The study was performed in accordance with the ethical codes of the World Medical Association (Declaration of Helsinki) and was approved by the local Ethics Committee (Comité de protection des personnes Ile de France XI, Ref 13005. CNIL, Ref DR-2013-283)

**Table 1:** Characteristics of the subjects who participated in the study. Control group data are shown at the bottom.

Patient	Time since stroke (years)	Age (years)	Gender	Type of stroke	Location of stroke
***1***	9	55	male	haemorrhagic	left temporo-parietal region
***2***	12	52	female	haemorrhagic	right temporal region
***3***	2	41	female	haemorrhagic	left temporo-parietal region
***4***	6	60	male	ischaemic	left complete mca* territory
***5***	12	56	male	haemorrhagic	left temporo-parietal cortex
***6***	9	45	male	ischaemic	right complete mca* territory
***7***	5	58	male	ischaemic	right complete mca* territory
***8***	5	28	female	ischaemic	left deep mca* territory
***9***	45	75	female	ischaemic	left complete mca* territory
***10***	4	59	male	ischaemic	left complete mca* territory
***11***	31	33	female	ischaemic	right superficial mca* territory
***12***	15	48	male	haemorrhagic	right temporal region
***13***	6	57	male	ischaemic	left superficial mca* territory
***14***	12	56	male	ischaemic	right superficial mca* territory
***15***	5	76	male	ischaemic	left superficial mca* territory
***16***	7	58	male	haemorrhagic	right fronto parietal region
***17***	9	34	female	haemorrhagic	right temporal region
***18***	9	61	male	ischaemic	right complete mca* territory
***19***	11	56	male	haemorrhagic	right fronto- parietal region
***20***	9	24	male	haemorrhagic	right fronto-parietal region
*Mean*	11.15 years	51.6±13.56			
*Summary Total*			n = 6 female n = 14 male	n = 9 haemorrhagic n = 11 ischaemic	n = 11 left hemisphere n = 9 right hemisphere
*Control Group*	-	49.7±11.7	n = 11 female n = 9 male	-	-

* mca: middle cerebral artery.

### 3.1 Study Design

The patients with stroke underwent tests of selected domains of cognitive ability before the TUG tests (see section 3.1.4). All subjects carried out the TUG and iTUG tests during a single assessment session. The order of the TUG tests (i.e. TUG followed by iTUG) was chosen to facilitate comparison with previous studies and to ensure that the patient correctly understood the task, since it involved several components [14,20,24]. Specifically, subjects performed two consecutive TUG tests then, after a break during which the iTUG was explained, they performed two consecutive iTUGs. MI ability was assessed using two validated versions of the Movement Imagery Questionnaire (MIQ). Although several methods of assessing MI exist (Guillot and Collet, 2005), temporal chronometry was chosen as much for its convenience as to facilitate comparison with existing research [14–17,20,24].

#### 3.1.1 The TUG test

All subjects were seated in a chair (with armrests), and on the verbal signal: “Ready-Set-Go” they were instructed to walk at a comfortable speed to a cone positioned three metres away, turn around the cone, walk back to the chair and sit down again. The time taken to complete this task was recorded in seconds using a manual stopwatch. The stopwatch was triggered after the verbal signal: “Ready-Set-Go” and was stopped when the patient's back contacted the back of the chair [1]. Two trials were recorded and averaged for each subject (as in the study design of Ayan et al., (2013) and Faria et al., (2009) [25,26]).

#### 3.1.2 iTUG test

The iTUG test was performed in the same conditions as the TUG [14]. Subjects were asked to remain seated in the chair, facing the track and cone. They could choose whether to close or open their eyes. The type of MI (KI or VI) was not imposed. The stopwatch was triggered after the same signal “Ready-Set-Go” and was stopped when patients said “Stop” after they had completed the imagined task (the task was considered complete when the subject imagined him/herself seated with his/her back in contact with the back of the chair). The exact instructions were: “When I tell you to start, you will imagine yourself performing the TUG. You will imagine yourself getting up from the chair, walking to the cone, turning around the cone, walking back to the chair sitting down again. I will say “ready-set-go” and you will start. Once you have imagined that you have sat down again, say “Stop”. You can keep your eyes open or closed. Two trials were recorded for each patient and averaged.

#### 3.1.3 Movement imagery questionnaire

The ability to perform an MI task was assessed using French versions of the Movement Imagery Questionnaire (MIQ). The revised (MIQ-R) version was used for the control group [27] and the revised second version (MIQ-RS) [28] was used for the patients with stroke. Both questionnaires contain questions relating to actions involving one or more limbs (lower and upper) as well as complex actions involving the whole body. The MIQ-RS [29] consists of fourteen questions relating to different movements. Patients rated the difficulty they had imagining the movements on a scale from 1 to 7 (where 1 was very difficult), and the maximum score was 98 [28]. The MIQ-R, used for the control subjects, consisted of eight questions with a maximum score of 56. The answers were also rated from 1 to 7 but concerned movements that could not be performed by patients with stroke which meant that although each test was validated within groups, test scores could not be directly compared between groups this explain why an intragroup percentage (of the total score for each MIQ test) was therefore calculated for intergroup comparison.

#### 3.1.4 Cognitive assessments (patients with stroke only)

We decided to not carry out cognitive tests in healthy controls because in the study of Beauchet et al., (2008) the group of older adults with cognitive impairment was aged on average 85.3 years whereas our control group was averaged 49.7 years. Moreover we compared our healthy subjects’ performances on temporal congruence to the results of other healthy subjects in other studies (Beauchet et al (2010) and Allali et al (2012)). Our healthy subject group has Standard Error very similar to that of the young healthy subjects of Beauchet et al (2010) and to the healthy subjects in the study by Allali et al (2012) who also used the iTUG (see Table A in S1 Appendix). In consequence, it appears unlikely that our control group had any cognitive disorders.

Three cognitive tests were chosen to assess the domains of executive function, global functions and attention/neglect. The tests were chosen as they are routinely used in clinical practice, are relatively rapid to perform (limiting fatigue-related bias) and results could be compared easily with previous research [24].

#### Assessment of executive functions

The French version [30] of the Frontal Assessment Battery at Bedside (FAB) [31] was used. It is composed of six sub-tests that assess conceptualization, mental flexibility, motor programming, environmental autonomy, interference sensibility and inhibitory control. Performance is rated from 0 (severe impairment) to 3 (normal) and a global score of less than 13/18 is considered pathological [31].

#### Global cognitive assessment

The Montreal Cognitive Assessment (MOCA) is a global assessment of cognitive function similar to the Mini Mental State Evaluation but is specific for patients with stroke. It is rated out of 30 and composed of seven sub-tests: denomination (rated from 0 to 3), memory (0 to 5), attention (0 to 6), language (0 to 3), abstraction (0 to 2) and orientation (0 to 6). A score of 0 denotes severe impairment and a score of less than 26 is considered pathological.

#### Assessment of attention and neglect

The Bells Test was used to assess visuospatial neglect and attention deficits [32,33]. Patients were given a sheet of A4 paper on which thirty-five bells were drawn (amongst other objects) and asked to cross out all the bells as quickly as possible. Time taken to finish the task, number of bells missed, and their position on the right or left of the paper were recorded. Time taken was adjusted for the patient’s age. The test was rated by counting missed bells. Six or more missed bells is considered abnormal. The position of missed bells provides an indication of unilateral spatial neglect [34].

### 3.2 Data Analysis

The 2 trials of the iTUG and TUG were averaged (± standard error) for each subject. Temporal congruence (± standard error) between the TUG and iTUG, was also calculated for each subject using the formula: [(TUG-iTUG)/((TUG+iTUG)/2)*100] and expressed as a percentage [14]. The formula was introduced by Bland & Altman (1986) to allow comparison with results from research conducted in real-world clinical practice [35,36]. Data were compared between groups (stroke patient vs controls) and within groups (executed vs imagined condition in the same group). The scores and sub scores of each cognitive test were calculated for each patient. In order to compare imagery ability between groups, the results of the MIQ-RS, the MIQ-R were expressed as a percentage of the total possible score.

Inter-group comparisons (stroke patient vs healthy controls) were carried out for TUG, iTUG and temporal congruence

### 3.3 Statistical Analysis

Normal distribution and homogeneity of the data were verified using the Shapiro Wilk procedure. Data were normally distributed except for TUG performance times in the patients with stroke. To verify our first hypothesis, the non-parametric Mann Whitney U test was used therefore to compare TUG, iTUG performances and temporal congruence between patients with stroke and controls; the sign test was used to compare the TUG and the iTUG within each group. The level of significance of the Mann Whitney U test and of the sign test were fixed at p < 0.05. To verify our second hypothesis, Spearman rank correlations were carried out between the scores of the cognitive tests and (i) iTUG performance and (ii) temporal congruence, to investigate the relationship between cognitive impairment and these two parameters in the patients with stroke. The significance of the correlation was set at p<0.016, following Bonferroni correction for the 3 types of cognitive assessments: FAB, MOCA and the Bells Test. All analyses were carried out using Statistica ® version 7.1 software.

## 4 Results

The characteristics of patients with stroke and healthy controls are shown in Table 1. There was no difference in the mean age of the two groups (p = 0.54).

### 4.1 Intergroup Comparisons

Results from the TUG and iTUG tests, temporal congruence (delta) and the MIQ percentages are summarized in Table 2.

**Table 2:** Mean (± Standard Error) TUG and iTUG performance times, temporal congruence and MIQ percentage values for both groups.

	PATIENTS WITH STROKE	CONTROL GROUP	P VALUE
**TUG (SECONDS)**	16.39±6.39	9.23±1.42	p<0.001*
**ITUG (SECONDS)**	10.56±0.56	7.33±1.76	0.001*
**TEMPORAL CONGRUENCE (%)**	45.11±35.11	24.36 ±17.91	0.02*
**MIQ-R(S) (%)**	80.25 ± 20.00	82.14 ±12.51	0.67
**KI (%)**	79.95±15.94	76.78±20.96	0.86
**VI (%)**	80.55±13.18	87.50±13.23	0.056

*Mann Whitney test, p < 0.05.

#### 4.1.1 TUG and iTUG test

Stroke patients performed the TUG test significantly more slowly than the control subjects (16.39±6.39s vs 9.23±1.42s, p<0.001). Stroke patients performed the iTUG test significantly more slowly than the control subjects (10.56 ±0.56s vs 7.33±1.76s, p = 0.01).

#### 4.1.2 Temporal congruence

Temporal congruence was significantly weaker in the patients with stroke than the control subjects (45.11±35.11% vs 24.36±17.91%, p = 0.02). A score of 0 indicates perfect congruence between the imagined and executed task, a score of 100 indicates a complete absence of congruence

#### 4.1.3 Movement imagery questionnaire

There was no significant difference on the global score (80.25±20.00% vs 82.14±12.51%, p = 0.67) neither in KI (79.95±15.94% vs 76.78±20.96%, p = 0.86) or in VI (80.55±13.18% vs 87.50±13.23%, p = 0.056) between the percentage MIQ assessment scores of the two groups.

### 4.2 Intragroup Comparisons

#### 4.2.1 Comparison of TUG / iTUG tests

Both groups performed the iTUG test significantly faster than the TUG test (respectively 10.56 ±0.56 vs 16.39 ±6.39, p = 0.003 for the patients with stroke and respectively 7.33 ±1.76 vs 9.23 ±1.42, p<0.001 for controls).

#### 4.2.2 Cognitive tests (patients with stroke)

Nine patients had a pathological global score (≤12) on the FAB executive functions assessment. The most impaired items were: Mental Flexibility, Motor Programming and Inhibitory Control.

Fifteen patients had a pathological score (≤26) on the Global Cognitive Function MOCA test. The most impaired functions were language and abstraction abilities.

Mean performance time for the Bells Test was 138.65 ±56.29s, and a mean 3.15 ±4.18 bells were missed. Regarding the location of the omissions, 1.6 ±2.41 were missed on the right-hand side and 1.55 ±1.93 were missed on the left-hand side, which did not indicate neglect. The results showed that seven patients had an attention disorder [34]. The results of the cognitive tests are summarized in Table 3.

**Table 3:** Cognitive Assessment Test Results.

Test	Sub-tests (mean score ± standard error)							
*Bells Test*	Time (sec)		Bells omitted		Right side omissions		Left side omissions	
	138.65 ±56.29		3.15 ±4.18		1.6 ±2.41		1.55 ±1.93	
*Montreal Cognitive Assessment (MOCA)*	global	Visuospatial/executive	Denomination	Memory	Attention	Language	Abstraction	Orientation
	22.05 ±5.05	3.7 ± 1.41	2.9 ± 0.3	2.5 ± 1.56	4.15 ± 1.52	1.8 ± 0.97	1.05 ± 0.49	5.65 ± 0.57
*Frontal Assessment Battery (FAB)*	global	Conceptualization	Mental Flexibility	Environmental Autonomy	Motor Programming	Sensitivity to interference	Inhibitory control	
	12.75 ±2.98	2.2 ± 0.87	1.4 ± 1.24	2.85 ± 0.65	1.5 ± 0.74	2.95 ± 0.21	1.85 ± 1.19	

Analysis of the Spearman’s correlations for cognitive test scores and (i) iTUG test times and (ii) temporal congruence (see Table 4) revealed a significant correlation between abnormal Bells Test results and longer iTUG test performance times (r = 0.85, p = 0.013). There were no other significant correlations.

**Table 4:** Spearman correlation analysis between iTUG, temporal congruence and cognitive test results (Bells, MOCA and FAB). For more details, see the text.

Cognitive Test	Sub-test	iTUG		Temporal Congruence	
		R	p	R	p
Bells test	Time	0.85*	0.013*	-0.60	0.14
	Omissions	-0.59	0.15	0.18	0.69
MOCA	Visuospatial/executive	0.43	0.09	-0.28	0.27
	Denomination	-0.04	0.88	-0.16	0.54
	Memory	-0.04	0.87	0.16	0.55
	Attention	0.11	0.66	0.02	0.93
	Language	-0.28	0.28	0.03	0.90
	Abstraction	-0.16	0.54	0.04	0.88
	Orientation	0.21	0.41	0.04	0.86
FAB	Conceptualization	0.55	0.09	-0.24	0.49
	Mental Flexibility	-0.12	0.73	0.49	0.14
	Environmental Autonomy	0.40	0.24	-0.29	0.41
	Motor Programming	0.57	0.08	-0.33	0.33
	Sensitivity to interference	-0.17	0.63	-0.17	0.63
	Inhibitory control	-0.50	0.13	0.04	0.90

* Significant correlation p <0.016.

All individual patients’ scores on cognitive and functional tests are available in Table B in S1 Appendix.

## 5 Discussion

This study is the first to explore temporal congruence between the TUG and iTUG in patients with stroke. Although a previous study explored gait related activities, they did not involve such complex tasks and did not use the same paradigm [22]. The results of the present study showed that: i) the patients with stroke performed both the iTUG and TUG significantly more slowly than the healthy controls; ii) temporal congruence was weaker in the patients with stroke (indicating reduced MI capacity) than healthy controls; iii) both the patients and healthy controls performed the iTUG significantly faster than the TUG, and iv) performance-time on the bells test was significantly correlated with the iTUG, suggesting that attentional disorders may affect MI capacity to perform the TUG test. These results confirm our first hypothesis that the MI capacity of patients with stroke would be reduced compared to healthy controls for the TUG task, and partially confirm our second hypothesis of a relationship between reduced MI capacity and cognitive impairment. Indeed, although the temporal congruence alterations was not correlated with cognitive disorders, it can be noticed that iTUG performance time was correlated with attentional impairment suggesting that MI is partially altered.

The finding that MI capacity was reduced in the patients is consistent with similar studies of imagined and executed motor tasks in patients with stroke [18,19,21,37]. However, the direction of the error between the imagined and actual movement differed from the results of previous studies that assessed the mental imagery of unilateral and voluntary movement in patients with stroke. For example, Malouin et al (2004, 2012) found that patients’ performance times were longer for imagined than executed movements [18,21]. However, our results are in accordance with previous studies of the iTUG carried out in other populations. Allali et al (2012) and Beauchet et al (2010) found shorter iTUG performance times than TUG performance times respectively in healthy controls and patients with multiple sclerosis and in young and older adults [13,22].

Differences in the direction of error of temporal congruence may reflect the task being evaluated. The present study investigated temporal congruence for a task that involved displacement of the whole body and was semi-automatic, whereas Malouin et al (2004, 2012) investigated the movement of a single limb during a voluntary task. It therefore seems that imagery performance time of a unilateral, voluntary task is longer than the executed task (as seen in Malouin et al (2004, 2012)) while imagery performance time of a global complex task is shorter than the executed task (as seen in Allali et al (2012) and Beauchet et al (2010)). This likely suggest that temporal congruence for simple upper or lower limb tasks does not necessarily predict the temporal congruence for functional gait tasks such as the TUG test.

Differences in the direction of the error in temporal congruence could also be attributed to the duration of the tasks, i.e. imagery performance time of lengthier tasks is shorter than executed performance time. Guillot & Collet (2005)also previously concluded that more complex motor tasks take longer to imagine [12]. In the present study, the patients with stroke took an average of 16 seconds to complete the TUG test, which is a relatively long task for MI. The task evaluated by Dettmers et al (2012) was of similar duration and was performed faster than the executed task, while Malouin et al (2004, 2012) assessed a task that lasted only four seconds and found that the imagined condition was performed more slowly than the real one [14,18,21,24].

The results of this study suggested also that cognitive impairment affects MI. Studies of other groups of patients with cognitive impairment (older people and patients with multiple sclerosis) have also demonstrated large error in temporal congruence mainly due to MI alterations [14,24]. This paper thus adds to that corpus by further identifying at least one of the types of cognitive dysfunction involved in disturbing MI in patients with stroke: impaired attention. In the present study, 15 patients had pathological MOCA scores, 9 had pathological FAB scores and 7 had pathological Bells Test scores. There were positive correlations between abnormally long times for completion of the Bells Test and longer performance times of the iTUG test, indicating that attention disorders may interfere with MI. These results are in accordance with those of Allali et al (2012) who found that attention deficits (as well as executive functions, and verbal fluency) were correlated with TUG and more particularly iTUG alterations responsible of errors in temporal congruence in patients with multiple sclerosis [24]. This relationship between attention and temporal congruence is probably highlighted by the fact that the task was lengthy. Comparison of the patients who exhibited attention disorders (n = 7) and those who did not (n = 13) revealed significant differences in temporal congruence (p = 0.014) (attention disorder: 75 ±36%, no attention disorder: 31 ±27%). Although these data are from a small sample, they suggest that the relationship between alterations in attention and disturbances in MI should be further investigated.

## 6 Limitations

There are a few points which might constitute limitations in the interpretations of the present data.

The fact that the subjects could freely choose their method of MI (KI or VI) for the iTUG could have interfered with the results since it has been shown that VI and KI activate different neural circuits [11]. This methodological choice was deliberated to allow subjects to spontaneously imagine and so help to ensure that their attention was focused on the task, and not diverted by the way they had to imagine it. We are, however, also reasonably confident that our results were not affected by differences in KI or VI as there was no statistical difference in the groups’ MI ability scores from the MIQ assessments.

**Fig 1:**
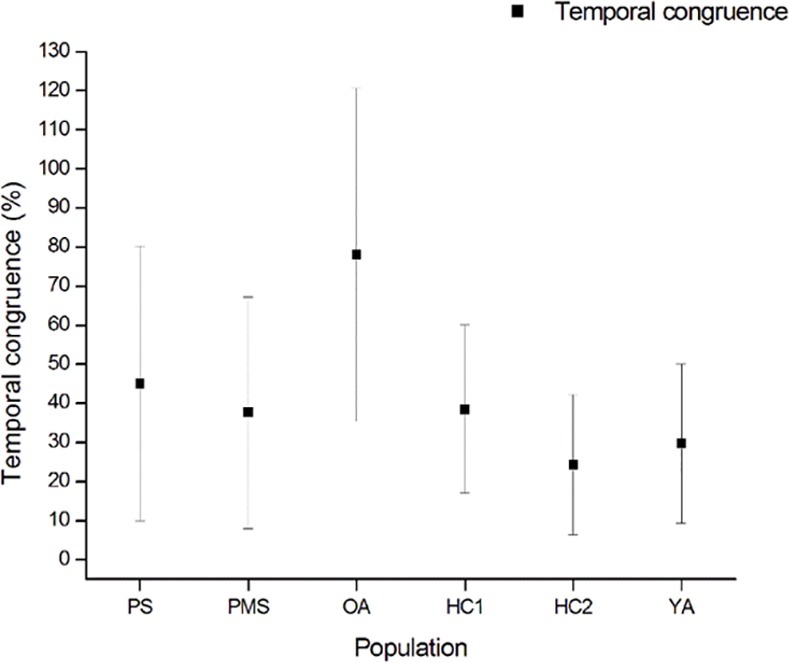
Comparison of temporal congruence between iTUG and TUG in patients with stroke (PS), patients with multiple sclerosis (PMS), older adults (OA), healthy controls 1 (HC1, Allali et al., (2012) study), healthy controls 2 (HC2, present study) and young adults (YA).

The results were based on the mean of two TUG and two iTUG tests, with a relatively large standard error. Although the Standard Error was quite large, it was also large in other studies assessing the iTUG: 37.70 ± 29.62% in patients with multiple sclerosis,38.52 ± 21.51% in healthy controls, 78.1 ± 42.6% in older adults and 29.73 ±20.3% in young adults [14,24] (Fig 1). This variability in all studies likely relies on the calculation method of temporal congruence.

To ensure that two trials were sufficient and did not introduce a biais in the interpretation of the results of the present study, we statically compared the data from the TUG1 vs TUG2 and the iTUG1 vs iTUG2 (additional analysis) in both groups by a Mann & Withney test. There is no significant difference between the two TUG (p = 0.82 in both group) and two iTUG (0.5 in control group and p = 0.11 in patients with stroke) in either groups so it seems that performing only 2 TUG and 2 iTUG test do not interfere with our results and that they can be interpreted with confidence (all the data are available in Table C in S1 Appendix). In addition this paradigm had already been used in other studies [25,26].

All subjects performed the TUG test before the iTUG test. This could constitute a confounding variable however was chosen to allow direct comparison of results with earlier studies performed in stroke as well as other patient populations [14,20,24].

## 7 Conclusion and Clinical Extrapolation

This study showed that the direction of alteration of temporal congruence depends on the task being assessed in both patients with stroke and healthy subjects. Disparities likely reflect differences in the nervous control of simple, voluntary movements compared with the control of complex tasks (semi-automatic and voluntary), however this remains to be tested using fMRI.

This study also showed that MI ability may be reduced in the case of cognitive impairment, particularly impairment of attention processes. There is modest evidence in the literature of improved effectiveness of rehabilitation in patients with stroke when MI is combined with traditional rehabilitation [38,39]. The results of the present study could explain the moderate effects of MI. We suggest that the use of MI for motor rehabilitation in patients with attention disorders should be adapted according to the presence and the severity of cognitive impairment, particularly attention deficits. Further studies should involve longitudinal evaluations of MI to determine whether changes in the capacity to imagine a motor task occur in parallel with changes in the ability to physically perform the task.

## Supporting Information

S1 Appendix **Table A**: Comparison of temporal congruence and standarStandard Error between iTUG and TUG in Healthy subjects in our study, in Beauchet et al (2010) and in Allali et al (2012). **Table B**: Individual patient' scores on cognitive and functional tests. **Table C**: Comparison of the two TUG and two iTUG in control and patients with stroke using the Mann & Withney test.
(DOCX)
