# Supplementary material for: Evaluating the Effect of Cognitive Dysfunction on Mental Imagery in Patients with Stroke Using Temporal Congruence and the Imagined ‘Timed Up and Go’ Test (iTUG)
Source: PLoS One. 2017 Jan 26;12(1):e0170400. doi: 10.1371/journal.pone.0170400 (PMC5268444; doi:10.1371/journal.pone.0170400)
Supplement: S1 Appendix — Table A: Comparison of temporal congruence and standarStandard Error between iTUG and TUG in Healthy subjects in our study, in Beauchet et al (2010) and in Allali et al (2012). Table B: Individual patient' scores on cognitive and functional tests. Table C: Comparison of the two TUG and two iTUG in control and patients with stroke using the Mann & Withney test. (DOCX) [file pone.0170400.s001.docx]

# Appendix

S1 Table A: Comparison of temporal congruence and standarStandard Error between iTUG and TUG in Healthy subjects in our study, in Beauchet et al (2010) and in Allali et al (2012).

| Group | Age | Mean Temporal Congruence | Standard Error of Temporal Congruence |
| --- | --- | --- | --- |
| Healthy Subjects in our study | 49.7±11.7 | 24.36 | 17.91 |
| Healthy Subjects in Beauchet et al (2010) | 25.7±2.3 | 38.52 | 21.51 |
| Healthy Subjects in Allali et al (2012) | 36.40±9.90 | 29.73 | 20.30 |

S1 Table B: Individual patient' scores on cognitive and functional tests.

| Patients | TUG | iTUG | MOCA | FAB | Bells test | | | |
| --- | --- | --- | --- | --- | --- | --- | --- | --- |
|  |  |  |  |  | Time (sec) | Bells omitted | Right side omissions | Left side omissions |
| 1 | 11.685 | 8.64 | 21** | 9** | 103 | 0 | 0 | 0 |
| 2 | 15.28 | 11.64 | 27 | 16 | 96 | 0 | 0 | 0 |
| 3 | 10.145 | 10.155 | 30 | 16 | 66 | 1 | 0 | 1 |
| 4 | 14.78 | 10.09 | 13** | 11** | 160 | 3 | 1 | 2 |
| 5 | 16.61 | 8.765 | 28 | 18 | 225** | 0 | 0 | 0 |
| 6 | 25 | 25.0475 | 28 | 17 | 157 | 0 | 0 | 0 |
| 7 | 8.81 | 5.86 | 22** | 13 | 82 | 0 | 0 | 0 |
| 8 | 13.33 | 8.395 | 24** | 16 | 104 | 2 | 0 | 2 |
| 9 | 14.81 | 2.81 | 22** | 12 | 175** | 3 | 2 | 1 |
| 10 | 26.215 | 13.375 | 19** | 8** | 123 | 0 | 0 | 0 |
| 11 | 15.14 | 12.965 | 10** | 11** | 250** | 3 | 2 | 1 |
| 12 | 13.64 | 7.55 | 22** | 12 | 180 | 9** | 6 | 3 |
| 13 | 14.31 | 5.23 | 20** | 14 | 64 | 16** | 8 | 8 |
| 14 | 15.705 | 17.17 | 18** | 13 | 150 | 1 | 0 | 1 |
| 15 | 25.14 | 14.175 | 16** | 9** | 207 | 5 | 3 | 2 |
| 16 | 39.715 | 16.125 | 25** | 12 | 236 | 1 | 0 | 1 |
| 17 | 15.595 | 15.03 | 27** | 12 | 104 | 2 | 0 | 2 |
| 18 | 11.825 | 6.94 | 23** | 14 | 109 | 7 | 5 | 2 |
| 19 | 11.985 | 5.315 | 20** | 7** | 101 | 10 | 5 | 5 |
| 20 | 8.13 | 6.06 | 26 | 15 | 81 | 0 | 0 | 0 |
| ** indicates a pathological score on a cognitive test | | | | | | | | |

S1 Table C: Comparison of the two TUG and two iTUG in control and patients with stroke using the Mann & Withney test.

| Group | TUG1 | TUG2 | p | iTUG1 | iTUG2 | p |
| --- | --- | --- | --- | --- | --- | --- |
| Control | 9.17±1.39 | 9.28±1.56 | **0.82** | 7.16±1.60 | 7.50±2.20 | **0.5** |
| Patients with stroke | 16.09±7.26 | 16.69±7.80 | **0.82** | 9.31±4.04 | 11.81±6.82 | **0.11** |
